# Polysulfide and Hydrogen Sulfide Ameliorate Cisplatin-Induced Nephrotoxicity and Renal Inflammation through Persulfidating STAT3 and IKKβ

**DOI:** 10.3390/ijms21207805

**Published:** 2020-10-21

**Authors:** Hai-Jian Sun, Bin Leng, Zhi-Yuan Wu, Jin-Song Bian

**Affiliations:** 1 Department of Pharmacology, Yong Loo Lin School of Medicine, National University of Singapore, Singapore 117597, Singapore; phcsunh@nus.edu.sg (H.-J.S.); lengbin@u.nus.edu (B.L.); 2 National University of Singapore (Suzhou) Research Institute, Suzhou 215123, China

**Keywords:** cisplatin, hydrogen sulfide, inflammation, STAT3, IKKβ

## Abstract

Cisplatin, a widely used chemotherapy for the treatment of various tumors, is clinically limited due to its extensive nephrotoxicity. Inflammatory response in tubular cells is a driving force for cisplatin-induced nephrotoxicity. The plant-derived agents are widely used to relieve cisplatin-induced renal dysfunction in preclinical studies. Polysulfide and hydrogen sulfide (H_2_S) are ubiquitously expressed in garlic, and both of them are documented as potential agents for preventing and treating inflammatory disorders. This study was designed to determine whether polysulfide and H_2_S could attenuate cisplatin nephrotoxicity through suppression of inflammatory factors. In renal proximal tubular cells, we found that sodium tetrasulfide (Na_2_S_4_), a polysulfide donor, and sodium hydrosulfide (NaHS) and GYY4137, two H_2_S donors, ameliorated cisplatin-caused renal toxicity through suppression of the massive production of inflammatory cytokines, including tumor necrosis factor α (TNF-α), interleukin-1β (IL-1β), interleukin-6 (IL-6), and cyclooxygenase-2 (COX-2). Mechanistically, the anti-inflammatory actions of Na_2_S_4_ and H_2_S may be mediated by persulfidation of signal transducer and activator of transcription 3 (STAT3) and inhibitor kappa B kinase β (IKKβ), followed by decreased phosphorylation of STAT3 and IKKβ. Moreover, the nuclear translocation of nuclear transcription factor kappa B (NF-κB), and phosphorylation and degradation of nuclear factor kappa B inhibitor protein alpha (IκBα) induced by cisplatin, were also mitigated by both polysulfide and H_2_S. In mice, after treatment with polysulfide and H_2_S donors, cisplatin-associated renal dysfunction was strikingly ameliorated, as evidenced by measurement of serum blood urea nitrogen (BUN) and creatinine levels, renal morphology, and the expression of renal inflammatory factors. Our present work suggests that polysulfide and H_2_S could afford protection against cisplatin nephrotoxicity, possibly via persulfidating STAT3 and IKKβ and inhibiting NF-κB-mediated inflammatory cascade. Our results might shed light on the potential benefits of garlic-derived polysulfide and H_2_S in chemotherapy-induced renal damage.

## 1. Introduction

Cisplatin is a widely used chemotherapeutic agent for the treatment of many solid organ cancers [1]. However, its clinical application for cancer therapy is largely limited because of its several adverse effects, including ototoxicity, neurotoxicity, and nephrotoxicity [2]. Among these side effects, nephrotoxicity is recognized to be most prevalent: accumulative evidence has revealed that over 30% of subjects might suffer from acute kidney injury after treatment with cisplatin [3]. Acute kidney injury is one of the most frequent complications in cancer patients upon cisplatin treatment [4]. As a consequence, the prevention and treatment of cisplatin nephrotoxicity remain a big challenge.

After decades of effort, the pathophysiological mechanisms of cisplatin nephrotoxicity have been identified. Upon cisplatin treatment, the renal proximal tubule cells are more vulnerable to cisplatin-induced cellular damage and later renal dysfunction, since proximal tubule cells have higher expressions of organic cation transporter 2, which may be helpful for them to uptake more cisplatin [5,6]. Mitochondrial dysfunction, reactive oxygen species (ROS) formation, caspase activation, and DNA damage are proposed to be involved in the pathologies of cisplatin-triggered acute kidney injury [7]. Moreover, the inflammation response is also suggested to be a major contributing factor for cisplatin-induced nephrotoxicity as proinflammatory cytokines in the kidneys are observed in significant numbers after administration of cisplatin [8]. Blockade of proinflammatory cytokines is reported to grant protection against cisplatin-induced nephrotoxicity [9]. Accordingly, anti-inflammatory compounds might serve as adjunctive therapies to prevent and treat cisplatin-related nephrotoxicity.

Recent studies have shed significant new lights on the potential strategies for the treatment of cisplatin nephrotoxicity, especially focusing on the renoprotective actions of phytochemicals [5,10]. Importantly, phytochemicals are well established to confer renoprotective effects against cisplatin-induced renal damage without affecting the anticancer efficacy of cisplatin [10,11]. Of these phytochemicals, garlic bioactive substances are found to effectively ameliorate cisplatin nephrotoxicity through anti-inflammatory and antioxidant properties [12,13]. The garlic derivatives are well-known folk remedies for various health issues due to its bioactive constituents, including polysulfide, diallyl trisulfide, and their potential as hydrogen sulfide (H_2_S) inducers [14,15,16]. It is believed that a comprehensive understanding of the roles of polysulfide and H_2_S in cisplatin nephrotoxicity will certainly support the use of garlic powders as renoprotective agents in the context of chemotherapy. Actually, we have previously demonstrated that both polysulfide and H_2_S not only grant renal protective effects in cisplatin-induced nephrotoxicity, but also greatly increase the anti-cancer sensitivity of cisplatin, suggesting that both of them could serve as attractive drugs for the management of cisplatin nephrotoxicity [17,18,19]. However, it remains to be investigated whether the protective effects of polysulfide and H_2_S on cisplatin-evoked renal damage are associated with the suppression of inflammatory cytokines. The present study was designed to explore the effects of polysulfide and H_2_S on cisplatin-elicited nephrotoxicity and renal inflammation. We expected that our present results would provide a basis for the further application of garlic bioactive substances in translational medicine treatment of cisplatin nephrotoxicity through the polysulfide and H_2_S signaling pathways.

## 2. Results

### 2.1. Polysulfide and H_2_S Attenuates Cisplatin-Induced Inflammation in Renal Tubular Cells through Inactivation of Signal Transducer and Activator of Transcription 3 (STAT3) and Inhibitor Kappa B Kinase β (IKKβ) Signaling

**Figure 1:**
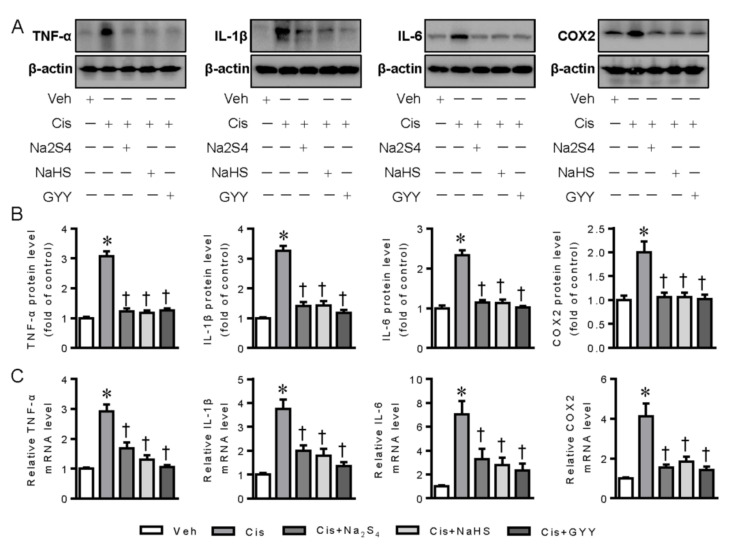
Polysulfide and H_2_S attenuated cisplatin-induced inflammation in renal tubular cells. (**A**) Representative blots of tumor necrosis factor α (TNF-α), interleukin-1β (IL-1β), interleukin-6 (IL-6), and cyclooxygenase-2 (COX-2). (**B**) Quantitative analysis of TNF-α, IL-1β, IL-6, and COX-2. (**C**) Relative mRNA levels of TNF-α, IL-1β, IL-6, and COX-2. Data were expressed as mean ± SEM. * *p* < 0.05 vs. Vehicle (Veh), † *p* < 0.05 vs. Cisplatin (Cis). GYY4137 (GYY). n = 4–6 in each group.

As renal tubular cell inflammation is closely associated with cisplatin nephrotoxicity (41), the possible roles of exogenous polysulfide and H_2_S donors in cisplatin-provoked inflammation were therefore examined in renal tubular cells. The results showed that pretreatment with polysulfide donor sodium tetrasulfide (Na_2_S_4_), H_2_S donors sodium hydrosulfide (NaHS), and GYY4137 clearly diminished the upregulated expressions of tumor necrosis factor α (TNF-α), interleukin-1β (IL-1β), IL-6, and cyclooxygenase-2 (COX-2) in renal tubular cells upon cisplatin challenge, as evidenced by Western blotting and RT-PCR analysis (Figure 1A–C).

**Figure 2:**
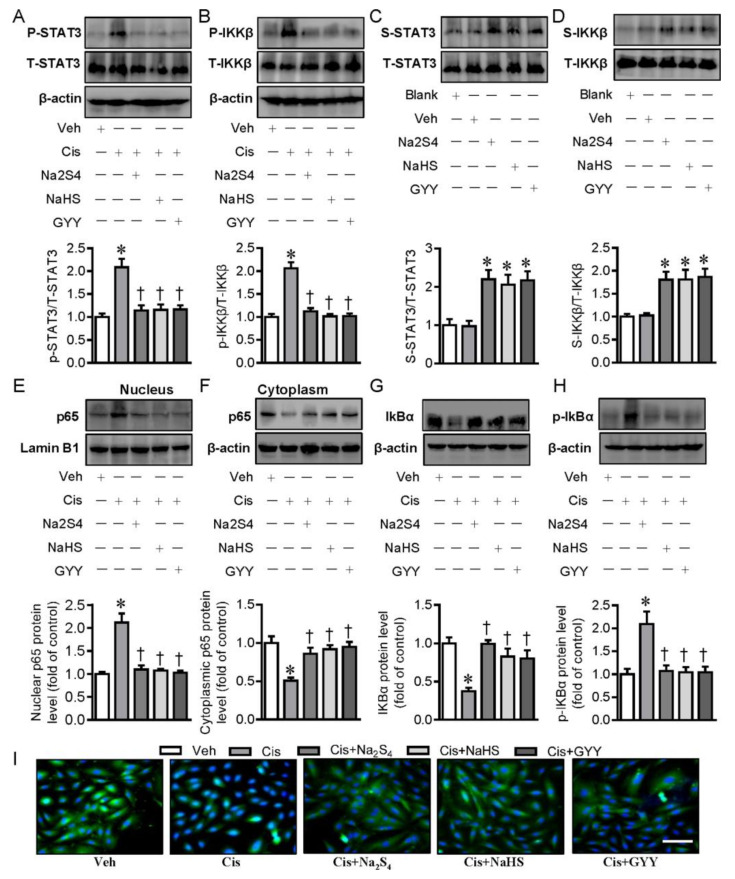
Both Na_2_S_4_ and NaHS attenuated the activation of transcription 3 (STAT3) and inhibitor kappa B kinase β (IKKβ) signaling in LLC-PK1 cells treated with cisplatin. (**A**) Representative blots of P-STAT3 (Phosphorylated STAT3). (**B**) Representative blots of P-IKKβ (Phosphorylated IKKβ). (**C**) Representative blots of STAT3 persulfidation. (**D**) Representative blots of IKKβ persulfidation. (**E**) Representative blots of nuclear p65 nuclear transcription factor kappa B (NF-κB). (**F**) Representative blots of cytoplasmic p65 NF-κB. (**G**) Representative blots of nuclear factor kappa B inhibitor protein alpha (IκBα). (**H**) Representative blots of P-IκBα (Phosphorylated IκBα). (**I**) Representative immunofluorescence images of p65 NF-κB. Scale bar = 50 μm. * *p* < 0.05 vs. Vehicle (Veh), † *p* < 0.05 vs. Cisplatin (Cis). GYY4137 (GYY). n = 4–6 in each group.

Subsequently, we examined the possible molecular mechanisms by which polysulfide and H_2_S exhibited anti-inflammatory characteristics in renal tubular cells. STAT3 and IKKβare two important transcription factors that play an orchestrated role in cisplatin-induced inflammation response and nephrotoxicity [20,21]. Recent studies have found that H_2_S could restrain the activation of STAT3 and IKKβ to alleviate inflammation [22,23]. Thus, we speculated that polysulfide and H_2_S may have anti-inflammatory effects through suppressing the activation of STAT3 and IKKβ in this experimental setting. As shown in Figure 2A,B, cisplatin led to the increased phosphorylation levels of STAT3 and IKKβ in renal tubular cells, and these increases were largely abrogated by pretreatment with polysulfide donor Na_2_S_4_, H_2_S donors NaHS, and GYY4137, further confirming that polysulfide and H_2_S were able to inhibit cisplatin-induced renal tubular cell inflammation through inactivation of STAT3 and IKKβ signaling.

Persulfidation (sulfhydration) of the specific cysteine residues within proteins is a chemical process that exerts a fundamental role in cellular redox regulation of polysulfide and H_2_S [24]. It has been found that H_2_S could inactivate IKKβ via persulfidating IKKβ at cysteine 179, thus inhibiting NF-κB activation and the subsequent inflammation response [23]. We recently reported that H_2_S directly induced the persulfidation of STAT3 at cysteine 259 and inactivated STAT3-mediated vascular calcification [25]. On the basis of these studies, we examined whether polysulfide and H_2_S inhibited the STAT3 and IKKβ signaling pathways via the persulfidation of them. When compared with control treatment, incubation with Na_2_S_4_ and H_2_S donors could significantly promote the persulfidation of STAT3 and IKKβ (Figure 2C,D), suggesting that the sulfhydryl group might be involved in the inhibition of STAT3 and IKKβ induced by either polysulfide or H_2_S.

### 2.2. Polysulfide and H_2_S Protects Renal Tubular Cells from Cisplatin-Induced Activation of NF-κB Signaling

Studies have shown that cisplatin induces the activation of NF-κB signaling, contributing to subsequent renal tubular cell inflammation and death [26]. The effects of polysulfide and H_2_S on NF-κB activation were examined thereafter. The obtained results showed that the nuclear translocation of NF-κB was observed in renal tubular cells upon cisplatin exposure, as reflected by Western blotting, suggesting NF-κB activation in the context of cisplatin (Figure 2E,F). Strikingly, polysulfide and H_2_S donors’ pretreatment significantly attenuated the nuclear translocation of NF-κB in renal tubular cells induced by cisplatin (Figure 2E,F). Furthermore, the enlargement, degradation, and phosphorylation of IκBα, events for NF-κB activation, were noticeably prevented by both polysulfide and H_2_S in cisplatin-treated cells (Figure 2G,H). Immunofluorescence results further confirmed that cisplatin-induced nuclear deposition of NF-κB in renal tubular cells was evidently prevented in the presence of polysulfide and H_2_S donors (Figure 2I).

**Figure 3:**
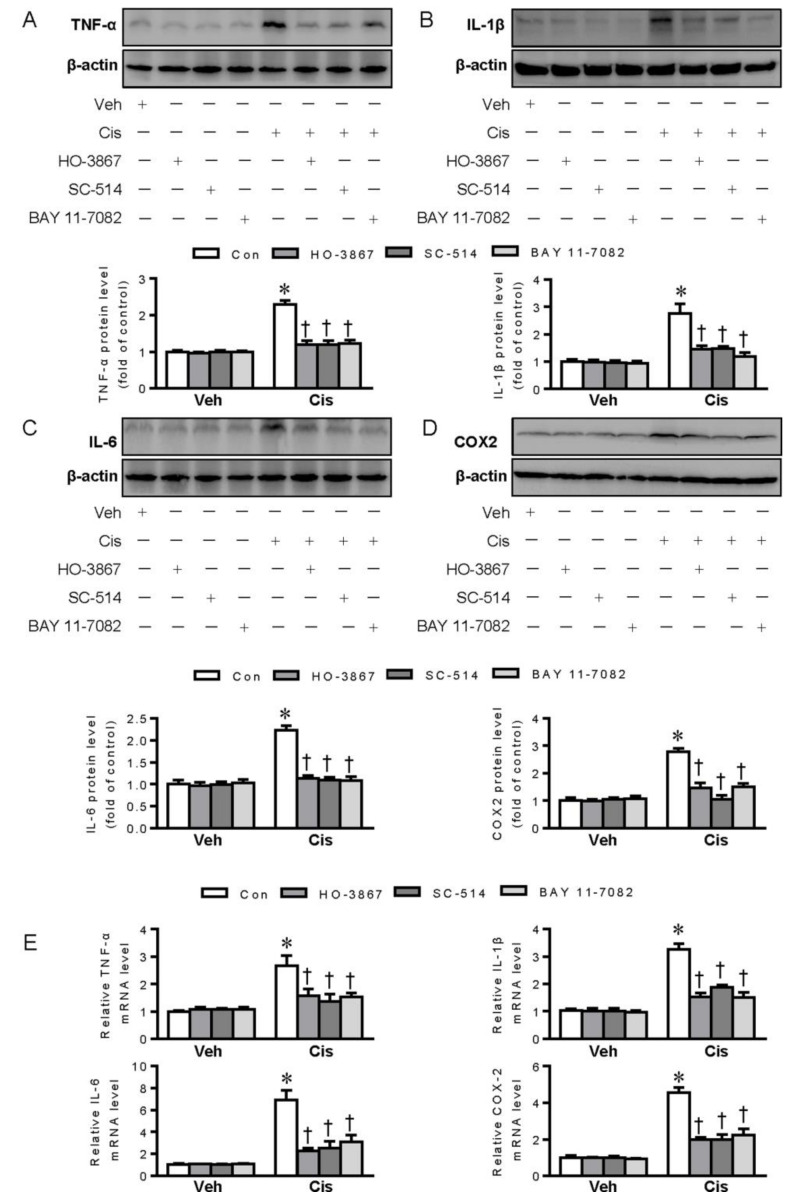
Pharmacological inhibition of STAT3, IKKβ, and NF-κB with their specific inhibitors prevented cisplatin (Cis)-induced inflammation in renal tubular cells. (**A**–**D**) Representative blots and quantitative analysis of TNF-α, IL-1β, IL-6, and COX-2. (**E**) Relative mRNA levels of TNF-α, IL-1β, IL-6, and COX-2. * *p* < 0.05 vs. Vehicle (Veh), † *p* < 0.05 vs. Control (Con). Cisplatin (Cis). n = 4–6 in each group.

Importantly, pharmacological inhibition of STAT3, IKKβ, and NF-κB with their specific inhibitors almost completely blocked the protein and mRNA expressions of TNF-α, IL-1β, IL-6, and COX-2 in cisplatin-incubated renal tubular cells (Figure 3A–E), indicating the critical involvement of STAT3/IKKβ/NF-κB signaling loop in cisplatin-mediated renal inflammation.

### 2.3. Polysulfide and H_2_S Ameliorates Cisplatin-Induced Renal Dysfunction and Inflammation in Mice

**Figure 4:**
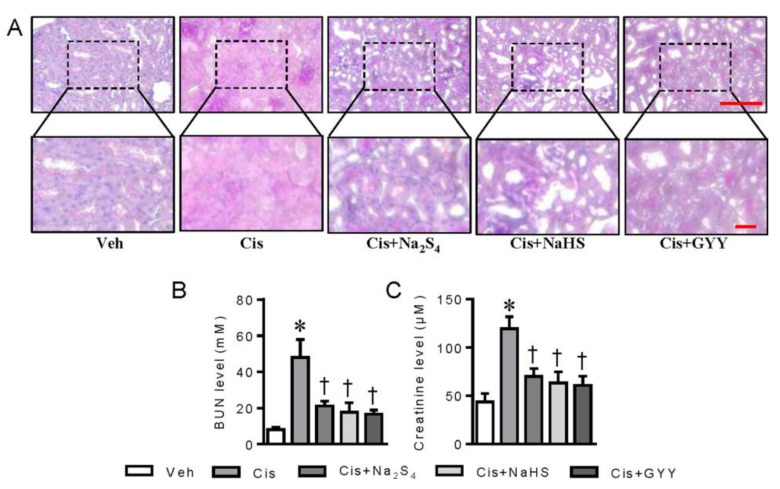
Polysulfide and H_2_S attenuated cisplatin-induced nephrotoxicity in mice. (**A**) Periodic acid-Schiff (PAS) staining of renal sections. Scale bar = 100 μm. (**B**) Serum blood urea nitrogen (BUN) levels. (**C**) Serum creatinine levels. * *p* < 0.05 vs. Vehicle (Veh), † *p* < 0.05 vs. Cisplatin (Cis). GYY4137 (GYY). n = 5 in each group.

To explore the renal protective effects of polysulfide and H_2_S, the pathological changes in renal tissues and renal function parameters were investigated in a mouse model of cisplatin nephrotoxicity. In agreement with our previous report [17], the renal sections of periodic acid-Schiff (PAS) staining exhibited obvious features of tubular injury in cisplatin-induced mice, while these pathological changes were obviously ameliorated by polysulfide donor Na_2_S_4_, H_2_S donors NaHS, and GYY4137 (Figure 4A). In line with the ameliorated renal morphology, the elevated serum BUN and creatinine levels in cisplatin-treated mice were remarkably reversed by both polysulfide and H_2_S donors (Figure 4B,C).

**Figure 5:**
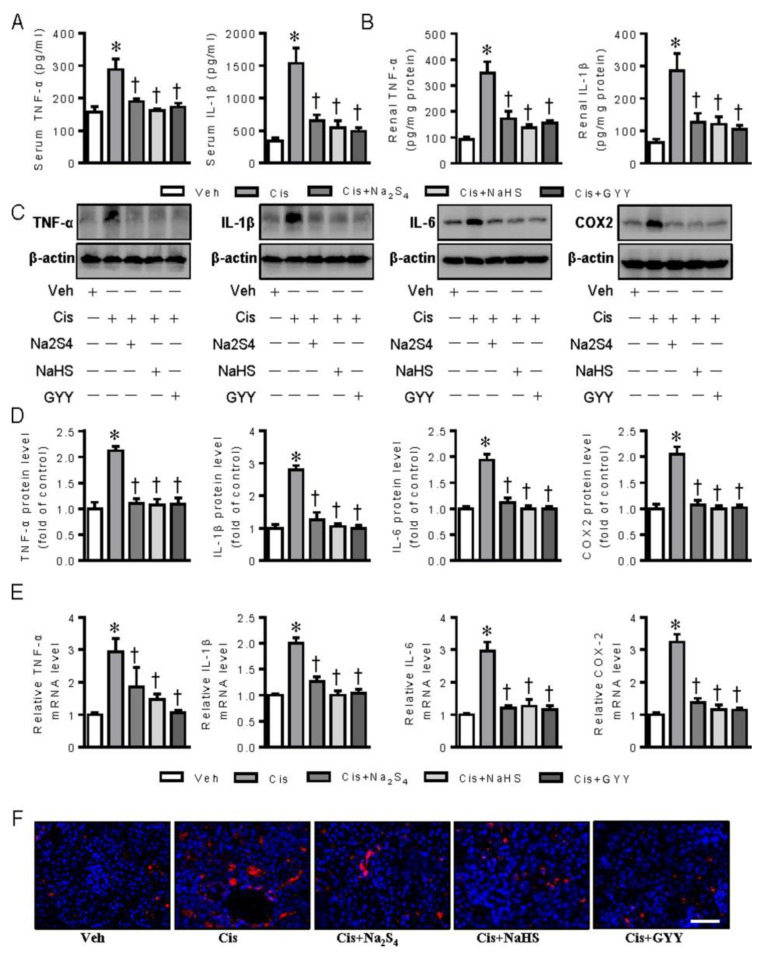
Polysulfide and H_2_S attenuated cisplatin-induced renal inflammation. (**A**) Serum levels of TNF-α and IL-1β. (**B**) Renal levels of TNF-α and IL-1β. (**C**) Representative blots of TNF-α, IL-1β, IL-6, and COX-2 in renal tissues. (**D**) Quantitative analysis of TNF-α, IL-1β, IL-6, and COX-2. (**E**) Relative mRNA levels of TNF-α, IL-1β, IL-6, and COX-2. (**F**) Representative immunofluorescence staining of macrophage marker F4/80. Scale bar = 20 μm. * *p* < 0.05 vs. Vehicle (Veh), † *p* < 0.05 vs. Cisplatin (Cis). GYY4137 (GYY). n = 5 in each group.

Moreover, the therapeutic effects of polysulfide and H_2_S on cisplatin-induced inflammation response were also assessed in mice as inflammation is critically involved in the pathologies of cisplatin nephrotoxicity [27]. As previously described [28,29], the levels of TNF-α and IL-1β in both serum and renal tissues were significantly enhanced in cisplatin-induced mice, and these upregulated pro-inflammatory cytokines were diminished by Na_2_S_4_, NaHS, and GYY4137 (Figure 5A–B). Then, we conducted RT-PCR and immunoblot to testify the expressions of pro-inflammatory cytokines in renal tissues, including TNF-α, IL-1β, IL-6, and COX-2. In the presence of Na_2_S_4_, NaHS, and GYY4137, the lower mRNA and protein levels of TNF-α, IL-1β, IL-6, and COX-2 in renal tissues were found when compared with the cisplatin group (Figure 5C–E). Macrophage marker F4/80 staining results further showed that both polysulfide and H_2_S could effectively attenuate cisplatin-induced renal inflammation (Figure 5F). These findings suggest an anti-inflammatory role of polysulfide and H_2_S in cisplatin nephrotoxicity.

### 2.4. Polysulfide and H_2_S Attenuates the Activation of STAT3, IKKβ, and NF-κB Signaling in Cisplatin-Induced Kidneys

**Figure 6:**
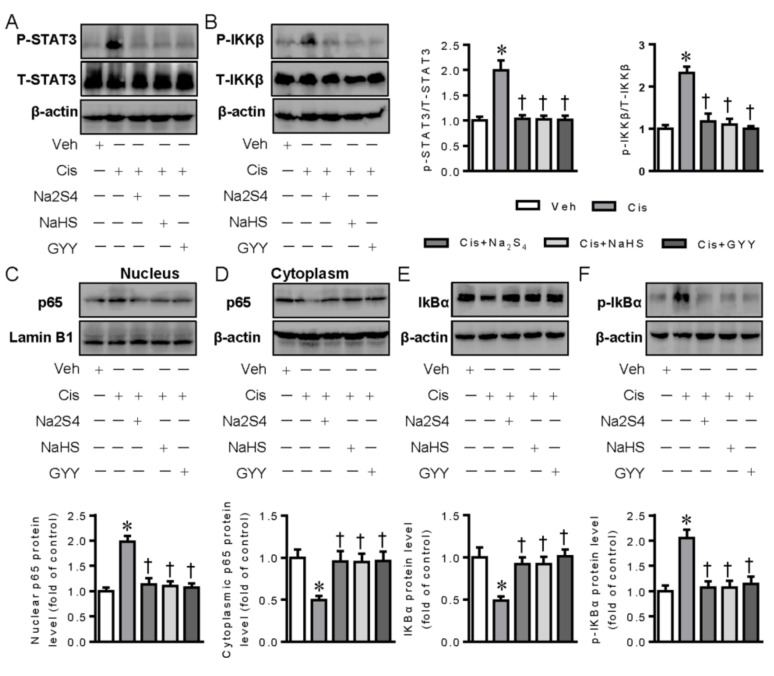
Polysulfide and H_2_S attenuated the activation of STAT3 and IKKβ in mice after cisplatin treatment. (**A**) Representative blots of P-STAT3. (**B**) Representative blots of P-IKKβ. (**C**) Representative blots of nuclear p65 NF-κB. (**D**) Representative blots of cytoplasmic p65 NF-κB. (**E**) Representative blots of IκBα. (**F**) Representative blots of P-IκBα. * *p* < 0.05 vs. Vehicle (Veh), † *p* < 0.05 vs. Cisplatin (Cis). GYY4137 (GYY). n = 5 in each group.

We further examined the effects of polysulfide and H_2_S on STAT3, IKKβ, and NF-κB activation in mouse renal tissues after administration of cisplatin. Similar to the results form cell experiments, cisplatin treatment led to higher phosphorylation levels of STAT3 and IKKβ in conjunction with NF-κB nuclear translocation in renal tissues, effects that were significantly mitigated by polysulfide and H_2_S donors (Figure 6A–D). Similarly, the phosphorylation and degradation of IκBα appeared to be higher in cisplatin-induced kidneys, which was markedly prevented by both polysulfide and H_2_S (Figure 6E,F). These results collectively indicate that polysulfide and H_2_S strongly inhibited STAT3/IKKβ/NF-κB-dependent release of pro-inflammatory cytokines in renal tissues from cisplatin-induced mice.

## 3. Discussion

Nephrotoxicity is one of the main side effects in cisplatin chemotherapy, thus limiting its clinical application for the treatment of cancer patients. This drives us to discover the novel reagents or chemicals that could prevent renal tubular cell injury induced by cisplatin without affecting the anti-tumor properties of cisplatin. As the two most abundant chemicals in garlic, polysulfide and H_2_S contribute to the clinical benefits of garlic derivatives in the context of various diseases [30,31]. Our present results demonstrated that polysulfide and H_2_S could be used for the management of cisplatin nephrotoxicity through inhibiting renal inflammation. Mechanistically, both polysulfide and H_2_S induced the persulfidation of STAT3 and IKKβ, two transcriptional factors involved in inflammation response [32], leading to STAT3 and IKKβ dephosphorylation. Moreover, the classical IKKβ-dependent NF-κB signaling pathway in cisplatin nephrotoxicity was also largely suppressed by administration of polysulfide and H_2_S donors. These results collectively demonstrated that both polysulfide and H_2_S could be employed as anti-inflammatory drugs to relieve cisplatin-induced nephrotoxicity. We also provide a possible clue that garlic’s organopolysulfide and H_2_S-releasing activity could promote health benefits through their strong anti-inflammatory activities.

The entrance of cisplatin into renal proximal tubular cells activates the apoptotic pathways and induces cellular damage via both oxidative stress and inflammation response [33]. The overproduction of inflammatory factors is one of the driving forces for renal proximal tubular cell death upon cisplatin treatment [34], while both polysulfide and H_2_S are well documented to exert anti-inflammatory effects [35,36]. We therefore examined the effects of polysulfide and H_2_S on cisplatin-induced inflammation response in renal proximal tubular cells. Our finding supported the notion that both polysulfide and H_2_S protected renal proximal tubular cells against inflammation response, as evidenced by measurement of TNF-α, IL-1β, IL-6, and COX-2. The direct anti-inflammatory effects of polysulfide and H_2_S may account for their anti-apoptotic effects on renal proximal tubular cells induced by cisplatin, and this had been demonstrated by our previous reports [17,19]. The protective effects of polysulfide and H_2_S were further demonstrated in animal models with cisplatin nephrotoxicity. We found that renal dysfunction and renal morphological changes in cisplatin-induced mice were markedly ameliorated by polysulfide and H_2_S donors together with attenuation of renal inflammation. The anti-inflammatory effects of polysulfide and H_2_S in animals were consistent with the cell culture experiments. Both in vivo and in vitro evidence suggested a potential of polysulfide and H_2_S in treating cisplatin nephrotoxicity associated with anti-inflammatory activities.

Thereafter, we explored the underlying mechanisms whereby polysulfide and H_2_S exerted anti-inflammatory effects on renal proximal tubular cells. The implications of STAT3 and IKKβ were examined because of their critical roles in the development of cisplatin-induced renal inflammation [37,38]. Similar to previous reports [20,39], cisplatin treatment led to higher phosphorylation levels of STAT3 and IKKβ, which were strikingly suppressed by polysulfide and H_2_S donors in renal proximal tubular cells. Importantly, inhibition of either STAT3 or IKKβ with their individual inhibitors attenuated cisplatin-induced upregulations of inflammatory factors, further confirming the critical engagement of the STAT3 and IKKβ pathway in cisplatin-induced renal inflammation.

We then investigated how polysulfide and H_2_S inhibited the phosphorylation of STAT3 and IKKβ in response to cisplatin. S-nitrosylation, a redox-based modification of protein thiols, has recently been recognized as nitric oxide (NO)-dependent cellular signal transduction mechanism [40], and it has been found that S-nitrosylation of STAT3 at cysteine 259 is crucial for inhibition of STAT3 transactivation-mediated inflammation [41]. Likewise, cysteine 179 within IKKβ is a main target for S-nitrosylation, thus contributing to anti-inflammatory effects of NO [42]. On these grounds, we hypothesized that polysulfide and H_2_S might lead to inactivation of STAT3 and IKKβ by the persulfidation of these proteins. The results showed that polysulfide and H_2_S donors obviously promoted the persulfidation of STAT3 and IKKβ using a tag-switch assay, indicating a possible interaction between organic sulfides and STAT3/IKKβ. These findings demonstrated that STAT3 and IKKβ are important targets for the redox regulation of polysulfide and H_2_S, thus providing a novel mechanism for their anti-inflammatory properties.

IKKβ is an important catalytic subunit for NF-κB activation, and NF-κB activation is involved in the upregulated expressions of various inflammatory cytokines and chemokines [43]. Indeed, cisplatin itself induces activation of NF-κB and its nuclear translocation is responsible for the production and release of inflammatory cytokines in renal proximal tubular cells [44]. The phosphorylation and degradation of IκBα is an upstream event for nuclear translocation and activation of NF-κB [45,46]. As such, interest in the treatment of cisplatin nephrotoxicity by targeting the NF-κB pathway has greatly increased due to its pathogenic roles in renal inflammation and damage. Here, we found that increased nuclear accumulation of NF-κB, phosphorylation, and degradation of IκBα were significantly inhibited by polysulfide and H_2_S donors in either cisplatin-treated renal proximal tubular cells or cisplatin-challenged kidneys from mice. These results collectively suggested that polysulfide and H_2_S granted a protection against cisplatin-induced nephrotoxicity through inhibiting NF-κB-mediated renal inflammation. This is in-keeping with a notion that both polysulfide and H_2_S are recognized as novel inhibitors of NF-κB [47,48].

In our previous reports [19], cisplatin administration led to a significant reduction in plasma H_2_S levels in animals, and this is in conjunction with reduced CSE expression in renal cortex tissues. Interestingly, the expression level of CBS in renal cortex remains unaltered in animals after administration of cisplatin [19]. Our previous findings suggest that administration of cisplatin results in the reduction of endogenous H_2_S production, which may be ascribed to the downregulated expressions of CSE in renal cortex [19]. In the present study, we demonstrated that polysulfide and H_2_S donors were effective in ameliorating renal dysfunction in a mouse model of cisplatin nephrotoxicity. It remains to be investigated whether the protective effects of polysulfide and H_2_S donors on cisplatin-induced renal damage are associated with the changes in plasma H_2_S levels or renal CBS/CSE expressions in mouse when treated with such donors. Therefore, the present investigation has limitations inherent to any integrative in vivo studies because the exact fate of polysulfide and H_2_S donors and their cellular interactions cannot be clarified. Regardless, it is necessary to measure the plasma H_2_S levels and renal CBS/CSE expressions in mice after administration of polysulfide and H_2_S donors, since such measurements are vital for a better understanding of their biological activities. In our future studies, the pharmacokinetics of polysulfide and H_2_S donors in animals and their effects on renal CBS/CSE expressions will be comprehensively explored.

After reviewing the published literatures, inconsistent results are observed with respect to the effects of H_2_S on cisplatin-induced nephrotoxicity. In a previously published paper [49], the authors found that application of GYY4137 further aggravated renal inflammation, oxidative stress, apoptotic response, and renal dysfunction in mice following cisplatin treatment. The discrepancy may result from the different preparation and dosages of GYY4137 used between the previous study and our present research. GYY4137 was prepared and stored in DMSO: PEG solution (1:1) by Liu and colleagues [49]. Nevertheless, it is well known that DMSO might accelerate the decomposition of GYY4137 [50]. Importantly, GYY4137 could be easily dissolved in water and a toxic solvent like DMSO might be unnecessary [50]. Moreover, Liu and colleagues used GYY4137 at a low dose of GYY4137 (21 mg/kg) in mice, and it is highly probable that H_2_S might be insufficiently provided at lower concentrations considering the H_2_S release characteristics of GYY4137 [51,52,53]. On these grounds, a commonly used dose of GYY4137 (100 mg/kg; dissolved in PBS) was prepared in our animal studies, and GYY4137 largely ameliorated cisplatin-induced renal dysfunction and damage, which is in-keeping with the results of NaHS (another H_2_S donor) application in animals [54,55,56]. These results clearly demonstrated the protective actions of H_2_S donors against cisplatin-induced nephrotoxicity.

Similar to the notion from Yang’s group [49], Francescato et al., have found that DL-propargylglycine (PAG), an inhibitor of endogenous H_2_S formation, reduces renal damage in rats induced by cisplatin [57]. In this study, an increased H_2_S generation rate and CSE expression were observed in the outer medulla from cisplatin-injected rats [57]. By contrast, exogenous H_2_S donors, NaHS, and sodium thiosulfate (STS) were found to protect against both cisplatin- and gentamicin-induced nephrotoxicity [54,58,59,60]. These conflicting results might be derived from a fact that PAG might be a non-specific inhibitor of CSE and also affects the functions of other enzymes that may play a central role in renal cellular responses to cisplatin. This viewpoint may be supported by the findings from the same group that treatment with increasing concentrations of NaHS had no effect on renal tubular cell survival, causing doubt about whether PAG is only inhibiting H_2_S production in this case [57]. Additionally, many studies have confirmed that endogenous H_2_S is protective against renal ischemia-reperfusion injury [61,62,63,64], renal fibrosis [65,66,67], and diabetic nephropathy [68,69]. In response to ischemia, H_2_S levels are diminished along with renal dysfunction, and inhibition of CBS leads to accumulation of homocysteine and renal ischemia-reperfusion injury [70,71]. In this regard, it is hard to give a clear unified view on these conflicting results, and the precise roles of endogenous or exogenous H_2_S in cisplatin-induced renal injury deserve further evaluation. Future research on the measurement of cellular sulfide levels during cisplatin-induced nephrotoxicity will be helpful to shed light on these contradicting findings. Furthermore, newly synthesized H_2_S donors or inhibitors will produce a better understanding of their exact roles as a renal protective molecule.

In conclusion, our findings suggest that the beneficial effects of polysulfide and H_2_S on cisplatin nephrotoxicity could be mainly due to their anti-inflammatory actions, especially in proximal tubular cells. The molecular mechanisms analysis showed that polysulfide and H_2_S ameliorated cisplatin nephrotoxicity by persulfidation and inactivation of STAT3 and IKKβ, as well as inhibition of NF-κB signaling. These results indicated that both polysulfide and H_2_S could serve as effective anti-inflammatory remedies for preventing and treating cisplatin nephrotoxicity. In light of the abundance of sulfur constituents in garlic, including polysulfide and H_2_S, this study may highlight the tremendous benefits of edible plants like garlic in cisplatin nephrotoxicity.

## 4. Materials and Methods

### 4.1. Reagents and Chemicals

Polysulfide donor Na_2_S_4_ was purchased from Dojindo Molecular Technologies Dojindo (Kumamoto, Japan). Cisplatin and NaHS were bought from Sigma-Aldrich (St. Louis, MO, USA). GYY4137 was synthesized as we previously depicted [72]. A selective and reversible inhibitor of IKKβ (IKK-2), SC-514, was procured from Santa Cruz Biotechnology Inc. (Santa Cruz, CA, USA). A selective STAT3 inhibitor HO-3867 was obtained from Medchem express (Princeton, NJ, USA). Antibodies against IL-6, COX-2, F4/80, Biotin, Lamin B1, and β-actin, and the secondary antibodies, were purchased from Santa Cruz Biotechnology Inc. (Santa Cruz, CA, USA). Antibodies against TNF-α, IL-1β, phosphorylated and total IκBα, NF-κB, phosphorylated and total STAT3, and phosphorylated and total IKKβ were obtained from Abcam (Cambridge, MA, USA). Dulbecco’s modified Eagle’s medium (DMEM), streptomycin/penicillin, trypsin, and fetal bovine serum (FBS) were provided by Hyclone Laboratories (South Logan, UT, USA). The radio-immunoprecipitation assay (RIPA) lysis buffer was purchased from Thermo Fisher Scientific Inc. (Waltham, MA, USA). Goat anti-mouse IgG (H+L) Highly Cross-Adsorbed Alexa Fluor 594 Secondary Antibody was procured from Invitrogen (Carlsbad, CA, USA). The specific primers used in this study were synthesized by Integrated DNA Technologies (Singapore).

### 4.2. Animals

All animal experimental procedures were approved by Institutional Animals Care and Use Committee at National of University of Singapore. During the process of animal experiments, the animals were subject to humane care in accordance with the criteria of the Care and Use of Laboratory Animal published by the US National Institutes of Health (National Institutes of Health publication, 8th edition, 2011). A total of 6–8 week male C57BL/6 mice were used to study the therapeutic effects of H_2_S and polysulfide on cisplatin nephrotoxicity. In detail, all mice were randomly divided into five groups (five mice for each group), including control group, cisplatin group, NaHS+cisplatin group, GYY4137+cisplatin group, and Na_2_S_4_+cisplatin group. NaHS (5.6 mg/kg, dissolved in normal PBS), GYY4137 (100 mg/kg, dissolved in PBS), and Na_2_S_4_ (500 μg/kg, dissolved in normal PBS) were administrated to mice by intraperitoneal injection a total of four times (once a day). After the second injection of these compounds for 30 min, cisplatin (25 mg/kg) was given by a single intraperitoneal injection. The control mice received normal PBS by intraperitoneal injection for 4 days. After the last time these agents were injected for 24 h, the blood samples and renal tissues were collected when animals were sacrificed. The blood samples and partial kidney tissues were stored at −80 °C for biochemical analysis. The remaining renal tissues were fixed in 4% paraformaldehyde for histology examination.

### 4.3. Histology Analysis and Immunofluorescence

To assess the renal morphology, the renal sections (5 μm) were subject to PAS staining. The stained sections were visualized under a light microscope (Leica, Microsystems, Germany). A total of six fields in each section were captured for the evaluation of pathological damage in-keeping with previous studies [28]. For immunofluorescent staining of F4/80, the renal sections were incubated with 5% serum for 60 min after dewaxing and rehydrating, and the sections were probed with anti-F4/80 antibody. The renal sections were then incubated with goat anti-mouse Alexa Fluor 594 antibody for 1 h at 37 °C. The nucleus was stained with 4′,6-diamidino-2-phenylindole (DAPI), and the images were obtained by a fluorescence microscope (Leica, Heidelberg, Germany).

### 4.4. Enzyme-Linked Immunosorbent Assay (ELISA)

The levels of TNF-α and IL-1β in the serum and renal tissues were measured by commercial ELISA kits (BOSTER, Wuhan, China) following the manufacturer’s protocols [73,74]. The renal levels of TNF-α and IL-1β were normalized to protein contents in each sample.

### 4.5. Assessment of Renal Function

At the end of animal experiments, the serum samples were collected and the serum BUN and creatinine levels were measured to evaluate kidney function by using commercially available kits (Nanjing Jiancheng Bioengineering Institute, Nanjing, China) in accordance with the manufacturer’s protocols [29,75]. The absorbance for serum BUN was analyzed at 640 nm by a microplate reader. The optical density was read at 546 nm for serum creatinine measurement using a Varioskan Flash microplate reader (Waltham, MA, USA).

### 4.6. Cell Culture and Treatment

LLC-PK1 cells, a porcine renal proximal tubular cell line (ATCC, Manassas, VA, USA), were cultured in DMEM containing 10% FBS, 1% penicillin, and streptomycin, under an environment with 95% air and 5% CO_2_ at 37 °C. After the cells achieved 80% confluence, the cells were passaged at a ratio of 1:3. To test the protective effects of H_2_S and polysulfide on cisplatin-induced renal toxicity, LLC-PK1 cells were pretreated with Na_2_S_4_ (80 μM), NaHS (100 μM), and GYY4137 (600 μM), for 30 min, and then were stimulated with cisplatin (30 μM) for 24 h. To examine the involvement of the STAT3 and IKKβ in cisplatin-trigged LLC-PK1 cell toxicity, HO-3867 (5 μM) and SC-514 (50 μM) were pre-added individually to the culture medium for 30 min before cisplatin (30 μM) treatment for 24 h.

### 4.7. Immunofluorescence Staining

After fixation and permeabilization, the cells were incubated with p65 NF-κB antibody overnight at 4 °C. The cells were then incubated with goat anti-mouse IgG (H + L) Highly Cross-Adsorbed Alexa Fluor 488 Secondary Antibody (1:200) for 30 min. Nuclei were counterstained with DAPI. Immunofluorescence signals were obtained using a fluorescence microscope (Leica, Heidelberg, Germany).

### 4.8. Western Blot Analysis

The dissected renal tissues or cultured cells were prepared for sequential extraction of proteins. The nuclear and cytoplasmic proteins were extracted as we previously described [17]. These samples were lysed in RIPA buffer, and the protein contents in each sample were determined by bicinchoninic acid (BCA) colorimetric protein kit (Pierce, MO, USA). The same amount of protein levels was loaded onto the sodium dodecyl sulfate polyacrylamide gel electrophoresis (SDS-PAGE) gels and then transferred to polyvinylidene fluoride (PVDF) membranes. After blocking in 5% skim milk at room temperature for 1 h, the membranes were probed with the required primary antibodies overnight at 4 °C. After that, these membranes were incubated with the corresponding secondary antibodies linked with horseradish peroxidase for 1 h. The positive bands on the membranes were visualized by enhanced chemiluminescence (ECL) substrate in BioRad ChemiDoc XRS system (Bio-RAD), and the density of the indicated bands was normalized to the levels of housekeeping gene on the same membrane.

### 4.9. S-Sulfhydration Assay

S-sulfhydration of STAT3 and IKKβ was measured by a tag-switch method [19,22]. In short, the collected cells were dissolved in HEN buffer (250 mM HEPES, 50 mM NaCl, 1 mM EDTA, 0.1 mM neocuproine, 1% NP-40). Cell lysates were incubated with a water-soluble methylsulfonyl benzothiazole (MSBT-A, 50 mM) at 37 °C for 1h. After salt removal, the mixture was added into anti-STAT3 antibody (2 μg) or anti-IKKβ antibody (2 μg) containing protein A/G beads overnight at 4 °C, respectively. The beads were then washed by PBS three times and resuspended in biotin-linked cyanoacetate (20 mM) at 37 °C for 1h under gentle shaking conditions. After centrifugation, biotinylated protein was pulled down by streptavidin magnet beads and eluted by SDS-PAGE loading buffer (30 μL), and the samples were further detected by Western blot. S-sulfhydrated STAT3 and IKKβ expressions were determined by using anti-biotin antibody.

### 4.10. Quantitative Real-Time PCR

After the isolation of total mRNA and cDNA synthesis, Synergy Brands Synergy Brands (SYBR) green-based (Promega, A6001) real-time PCR amplification was conducted under real-time PCR system (Applied Biosystems, Carlsbad, CA, USA) according to the manufacturer’s instructions. The mRNA levels of the target genes were quantified using the 2^−ΔΔCt^ method and normalized to the expression of glyceraldehyde 3-phosphate dehydrogenase (GAPDH). The specific sequences of used primers in the present study were listed in Tables S1 and S2.

### 4.11. Statistical Analysis

The results were expressed as mean ± SEM. Unpaired *t*-test was used to compare one variable within two groups. ANOVA was applied to compare one variable in three or more groups followed by Bonferroni’s multiple comparisons test. Differences with a *p*-value of less than 0.05 were predetermined as the threshold for statistical significance.

## Supplementary Materials

Supplementary Materials can be found at https://www.mdpi.com/1422-0067/21/20/7805/s1.

## Abbreviations

BUN	blood urea nitrogen
COX-2	cyclooxygenase-2
DAPI	4′,6-diamidino-2-phenylindole
ELISA	enzyme-linked immunosorbent assay
FBS	fetal bovine serum
H_2_S	hydrogen sulfide
IL-1β	interleukin-1β
IL-6	interleukin-6
IKBα	nuclear factor kappa B inhibitor protein alpha
IKKβ	inhibitor kappa B kinase β
NaHS	sodium hydrosulfide
NF-κB	nuclear transcription factor kappa B
NO	nitric oxide
PAG	DL-propargylglycine
PAS	periodic acid-Schiff
ROS	reactive oxygen species
STAT3	signal transducer and activator of transcription 3
STS	sodium thiosulfate
TNF-α	tumor necrosis factor α
