# Supplementary material for: Polysulfide and Hydrogen Sulfide Ameliorate Cisplatin-Induced Nephrotoxicity and Renal Inflammation through Persulfidating STAT3 and IKKβ
Source: Int J Mol Sci. 2020 Oct 21;21(20):7805. doi: 10.3390/ijms21207805 (PMC7589167; doi:10.3390/ijms21207805)
Supplement: Supplementary file 1 [file ijms-21-07805-s001.pdf]

Table S1. Primers for real-time quantitative PCR analysis in pork cells.

	Primer	Sequence
TNF-α	Forward	5′-CCCCCAGAAGGAAGAGTTTC-3′
	Reverse	5′-CGGGCTTATCTGAGGTTTGA-3′
IL-1β	Forward	5′-GAAATGGGAGCATCCAGCTGCAAA-3′
	Reverse	5′-TTGCACGTTTCAAGGATGATGGGC-3′
IL-6	Forward	5′-ATGCTCTTCACCTCTCCGGACAAA-3′
	Reverse	5′-TTCTGCCAGTACCTCCTTGCTGTT-3′
COX-2	Forward	5′-TGTGAAAGGGAGGAAAGA-3′
	Reverse	5′-CTGATGGGTGAAGTGCTG-3′
GAPDH	Forward	5′-ACCCAGAAGACTGTGGATGG-3′
	Reverse	5′-ACGCCTGCTTCACCACCTTC-3′

Table S2. Primers for real-time quantitative PCR analysis in mice.

	Primer	Sequence
TNF-α	Forward	5′-CACCATGAGCACAGAAAGCA-3′
	Reverse	5′-TAGACAGAAGAGCGTGGTGG-3′
IL-1β	Forward	5′-TGGACCTTCCAGGATGAGGACA-3′
	Reverse	5′-GTTCATCTCGGAGCCTGTAGTG-3′
IL-6	Forward	5′-CAAAGCCAGAGTCCTTCAGAG-3′
	Reverse	5′-GCCACTCCTTCTGTGACTCC-3′
COX-2	Forward	5′-TTAAGGCATCACAGTCCGAG-3′
	Reverse	5′-TTGAATGTGAAGTTGACCCG-3′
GAPDH	Forward	5'-AGGTTGTCTCCTGCGACTTCA-3'
	Reverse	5'-TGGTCCAGGGTT TCTTACTCC-3'
